# Materials characterization of TiO_2_ nanotubes decorated by Au nanoparticles for photoelectrochemical applications

**DOI:** 10.1039/d1ra07443a

**Published:** 2021-12-02

**Authors:** Marcin Pisarek, Mirosław Krawczyk, Andrzej Kosiński, Marcin Hołdyński, Mariusz Andrzejczuk, Jan Krajczewski, Krzysztof Bieńkowski, Renata Solarska, Magdalena Gurgul, Leszek Zaraska, Wojciech Lisowski

**Affiliations:** Institute of Physical Chemistry, Polish Academy of Sciences Kasprzaka 44/52 01-224 Warsaw Poland mpisarek@ichf.edu.pl +48 22 343 3333 +48 22 343 3325; Faculty of Materials Science and Engineering, Warsaw University of Technology Wołoska 141 02-507 Warsaw Poland; Faculty of Chemistry, University of Warsaw Pasteura 1 02-093 Warsaw Poland; Laboratory of Molecular Research for Solar Energy Innovations, Centre of New Technologies University of Warsaw Banacha 2c 02-097 Warsaw Poland; Faculty of Chemistry, Jagiellonian University in Kraków Gronostajowa 2 30-387 Kraków Poland

## Abstract

The structural and chemical modification of TiO_2_ nanotubes (NTs) by the deposition of a well-controlled Au deposit was investigated using a combination of X-ray photoelectron spectroscopy (XPS), scanning electron microscopy (SEM), Scanning Transmission Electron Microscopy (STEM), Raman measurements, UV-Vis spectroscopy and photoelectrochemical investigations. The fabrication of the materials focused on two important factors: the deposition of Au nanoparticles (NPs) in UHV (ultra high vacuum) conditions (1–2 × 10^−8^ mbar) on TiO_2_ nanotubes (NTs) having a diameter of ∼110 nm, and modifying the electronic interaction between the TiO_2_ NTs and Au nanoparticles (NPs) with an average diameter of about 5 nm through the synergistic effects of SMSI (Strong Metal Support Interaction) and LSPR (Local Surface Plasmon Resonance). Due to the formation of unique places in the form of “hot spots”, the proposed nanostructures proved to be photoactive in the UV-Vis range, where a characteristic gold plasmonic peak was observed at a wavelength of 580 nm. The photocurrent density of Au deposited TiO_2_ NTs annealed at 650 °C was found to be much greater (14.7 μA cm^−2^) than the corresponding value (∼0.2 μA cm^−2^) for nanotubes in the as-received state. The IPCE (incident photon current efficiency) spectral evidence also indicates an enhancement of the photoconversion of TiO_2_ NTs due to Au NP deposition without any significant change in the band gap energy of the titanium dioxide (*E*_g_ ∼3.0 eV). This suggests that a plasmon-induced resonant energy transfer (PRET) was the dominant effect responsible for the photoactivity of the obtained materials.

## Abstract 2

The structural and chemical modification of TiO_2_ NTs by the deposition of a well-controlled Au deposit (0.01 mg cm^−1^) was investigated using a combination of microscopic (SEM, STEM), analytical measurements (XPS, SERS, UV-Vis, XRD) and photoelectrochemical investigations.

## Graphical abstract

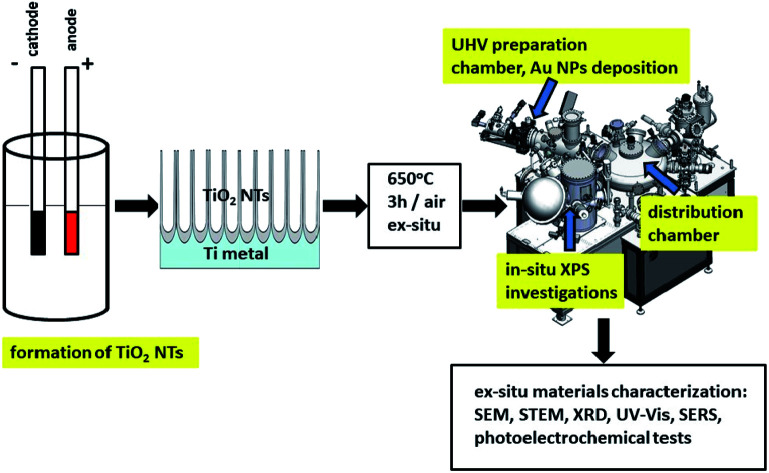

## 1. Introduction

The decoration of TiO_2_ nanostructures with noble metal nanoparticles (NPs) such as Au is one way to improve photocatalytic activity,^1^ and so such nanostructures are still considered promising materials for solar energy conversion and photocatalytic performance.^2,3^ Gold is well known for being chemically inert towards oxidation, which makes photocatalysts modified by Au NPs more stable and durable in photocatalytic reactions. Moreover, Au NPs have unique electronic, optical, and magnetic properties, such as the strong absorption of visible light due to their plasmon resonance (SPR) effect.^4^ Noble metal NPs in close contact with TiO_2_ form Schottky barriers, which drive photogenerated electrons from the n-type TiO_2_ to the noble metals and enhance the charge separation rate, as well as the photocatalytic activity. For TiO_2_ decorated with Au NPs, an additional effect occurs: the localized surface plasmon resonance (LSPR), which contributes to a strong absorption of visible light and therefore also to improved photocatalytic performance under visible light illumination.^5^ Hence, the quality of the Schottky junctions plays a crucial role in the process of photoexcited carrier separation.^6^ It is generally accepted that, depending on the electronic structure of the semiconductor substrate, LSPR can either promote hot electron transfer from plasmonic NPs to the semiconductor conduction band (CB) upon crossing the Schottky barrier junction, or result in a plasmon-induced resonant energy transfer (PRET).^1^ A higher electromagnetic field generates more hot electrons. Therefore, the LSPR effect is readily affected by the shape, size, and number of Au NPs, and by the characteristics of the TiO_2_ supports.^1^ So, this kind of materials are one of the most important classes of heterogeneous catalysts, and are widely employed in the chemical industry and in environmental protection.^7^ Superior catalytic performance is usually related to what is known as the strong metal support interaction (SMSI) between metal NPs and metal oxide supports.^8–10^ Goodman^11,12^ explained the SMSI effect for catalytically active Au on titania. He showed that, for an active Au/TiO_2_ model catalyst, the core level shifts measured by X-ray photoelectron spectroscopy (XPS) are consistent with the electron transfer from titania to Au leading to electron-rich Au, as has been confirmed by density functional calculations. The large number of publications on plasmon-driven photocatalysis that have already appeared mainly they report on the idea of preparing photoactive materials having a precisely designed electronic structure or a morphology based on deposition methods of Au NPs.^4,5,13^ Moreover, using TiO_2_ NTs as a support for Au NPs seems to be a good idea for obtaining nanostructures suitable for photocatalytic/electrophotocatalytic/catalytic applications. This is because such oxide nanomaterials are distinguished by a well-defined geometry (diameter, length, wall thickness of the nanotubes), a uniform chemical composition, and a crystalline structure that can be controlled by heat treatment and modification of the electronic structure (TiO_2_/p-type semiconductor interfaces forming p–n junctions).^14–16^ Various methods of depositing Au NPs on the tops of nanotubes have been proposed. Usually, chemical/electrochemical methods^6,17,18^ assisted by photo-reduction or microwave chemical reduction processes are applied.^19^ The precursor of gold was usually chloroauric acid (HAuCl_4_). These methods lead to the formation of spherical gold nanoparticles of various sizes ranging from 5 to 50 nm. In addition, the preparation of Au NPs based on evaporation and sputtering processes has also been reported.^20,21^ The nanostructures thus prepared proved to be effective photo-catalysts for the decomposition of a methylene blue (MB) solution under visible light irradiation,^13,19^ as well as for the degradation of toluene,^20^ rhodamine B,^22^ methyl orange^23^ and the decomposition of acid orange 7.^21^ These kinds of materials were also used for photocatalytic H_2_ production^24^ and CO_2_ conversion to methane (CH_4_).^25^ This is due to the local formation of Schottky junctions (electron transfer) and the LSPR effect, resulting in a significant enhancement in the photoresponse of TiO_2_ NTs in the visible range due to the presence of Au NPs. The above literature data show that materials decorated with Au nanoparticles are effective photoactive materials. An important feature of these materials is the ability to create surface geometries by controlling the size of the nanotubes during the forming process,^14,26,27^ and thus the distribution of plasmonic nanoparticles on their surface. Then the nanoparticles are located not only at the edges of the nanotubes but also on their inner and outer walls. Thanks to this the highly ordered structure of nanotubes, the directional movement of photogenerated charges (along the axis of the tubes) to the metallic titanium substrate occurs as easily as possible, with the result that, photoelectrodes having a well-defined morphology, structure and good mechanical stability are obtained.^28^ Moreover, TiO_2_ decorated with gold nanoparticles can ensure efficient charge separation, *i.e.* recombination of electron–hole pairs, which leads to increased photocatalytic test reactions.^14,22^ It is very important, therefore, to obtain plasmonic nanoparticles of a specific size and dispersion on the surface of the photomaterials. This makes it possible to apply the thermal evaporation method by using an Au effusion cell under ultra-high vacuum conditions, where the factor controlling the size of nanoparticles is the deposition rate. In the present work, we show for the first time the preparation and electronic properties of TiO_2_ NTs surface-modified by ultra-high vacuum sputtering of Au NPs at a much lower rate (0.03 nm min^−1^). This preparation method leads to the formation of unique photoactive materials. The mechanism of formation of photo-active centers in such nanomaterials, where the SMSI and LSPR effects play a crucial role, is discussed in this paper as well.

## 2. Experimental methods

**Fig. 1:**
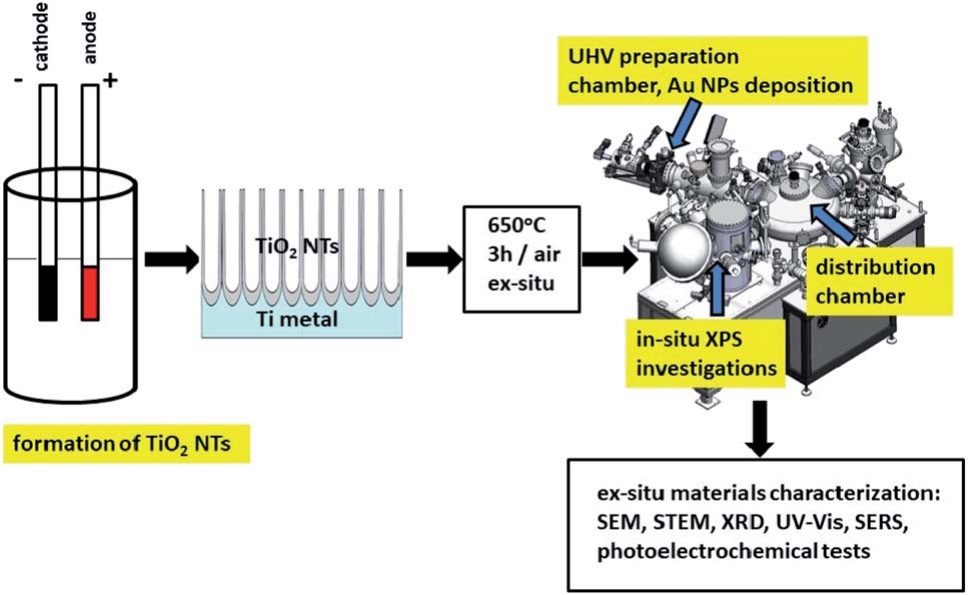
Schematic diagram of sample preparation and characterization.

Fig. 1 shows a schematic diagram of experimental set up for the synthesis and characterization of the fabricated materials. Details of the investigation are given below.

### 2.1 Sample preparation

TiO_2_ nanotubes (NTs) were fabricated *via* a one-step anodization process on Ti foil (0.25 mm-thick and 99.5% + % purity – Alfa Aesar) at 25 V for 3 h in an optimized electrolyte based on glycerol and water mixture (volume ratio 50 : 50) containing 0.27 M ammonium fluoride (NH_4_F) by using a two-electrode electrochemical system. After anodization, the samples were rinsed with DI water (24 h) and dried in air. Subsequently, thermal annealing in air was performed at 650 °C for 3 h.

Gold nanoparticles were deposited by thermal evaporation using the EF 40C1 effusion cell inside the UHV preparation chamber (PREVAC, Rogów, Poland). The cell was maintained at a temperature of 1150 °C during the process of resistive evaporation. The gold foil (0.1 mm-thick and 99.9975 + % purity, Alfa Aesar) was evaporated onto the room temperature-surface of TiO_2_ nanotubes at a pressure of 1–2 × 10^−8^ mbar. The average amount of metal deposited per cm^2^ (∼0.010 mg cm^−2^) was strictly controlled, as was the mass gain of the samples (measured with a quartz microbalance – TM-400 (Maxtek Inc.)) during the metal deposition process at a constant evaporation rate of 0.03 nm min^−1^. After deposition of the gold NPs, the TiO_2_ nanotubes samples were then transferred to the XPS analysis chamber.

### 2.2 Surface and structure characterization

The chemical composition and chemical state of the prepared sample were characterized by X-ray photoelectron spectroscopy (XPS). For this purpose, a PHI 5000 VersaProbe (ULVAC-PHI) spectrometer was used. The XPS spectra were excited using Al_Kα_ (*hν* = 1486.6 eV, 25 W) monochromatic radiation as a source, at a resolution of binding energy 0.1 eV. The survey and high-resolution (HR) spectra were collected at a constant pass energies of 117.4 and 23.5 eV, respectively. Avantage Surface Chemical Analysis software – ThermoFisher Scientific (ver. 5.9911) was used for the data processing. The background was corrected using the Smart model to obtain the XPS signal intensity. An asymmetric Gaussian/Lorentzian function at a constant ratio G/L = 0.35 was used for the deconvolution procedure. The measured binding energies for individual elements were corrected in relation to the C 1s carbon peak at 284.8 eV.

For the morphological characterization of the samples after their anodization, heat treatment and functionalization in the vacuum preparation chamber, examinations were carried out under a high vacuum (pressure 10^−7^ mbar) with a scanning electron microscope (FEI Nova NanoSEM 450) by using the secondary electron detector (SE-TLD). Images were obtained at a long scan acquisition time (20 μs) of typically 30 seconds per frame after choosing the inspection region.

Microstructure and structural investigations were performed using a Hitachi HD-2700 high-resolution scanning transmission electron microscope (HR-STEM) operating at 200 kV. The TEM examinations were performed on thin samples prepared by a Hitachi NB5000 focused ion beam (FIB) system. The samples were prepared as cross-sections of the oxide layers with the gold deposits in order to clearly see the structure of the metal nanoparticles. After FIB preparation, the lamellas were finally thinned using low-energy argon ion milling on a Gentle Mill (Technoorg Linda Ltd). In addition, STEM microscopic images were used for the image analysis to determine the size distribution of the Au NPs based on ImageJ software.^29^

X-ray powder diffraction data were collected on a PANalytical Empyrean diffractometer fitted with a X'Celerator detector using Ni-filtered Cu Kα radiation (*λ*_1_ = 1.54056 Å and *λ*_2_ = 1.54439 Å). Data were collected on a flat plate *θ*/*θ* geometry on a spinning sample holder. All presented data were collected in the 2*θ* range 10–90°, in intervals of 0.0167°, with a scan time of 30 s per interval.

Raman measurements were carried out using a Horiba Jobin-Yvon Labram HR800 spectrometer equipped with a Peltier-cooled CCD detector (1024 × 256 pixel), a 600 grooves/mm holographic grating, and an Olympus BX40 microscope with a long distance 50× objective. A diode pumped, frequency doubled Nd:YAG laser provided the excitation radiation with the wavelength of 532 nm.

UV-Vis absorbance spectra were collected using a Jasco V-650 spectrophotometer equipped with a 60 mm diameter integrating sphere. Bandgap calculations were performed from the diffusion spectra using the built-in software with the Kubelka–Munk model. The Kubelka–Munk equation (eqn (1)) describes the relationship between diffused reflectance and the absorption/diffusion coefficient.1
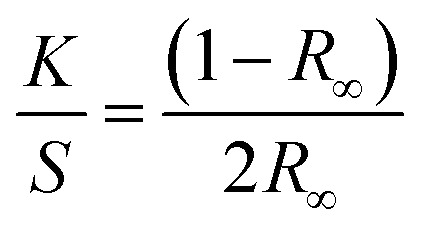
where *K* is the absorption coefficient, *S* is the diffusion coefficient, and *R*_∞_ is reflectance when the sample thickness makes the effect of the base negligible.

### 2.3 Photoelectrochemical characterization

The photoelectrochemical characterization of the samples was performed using a photoelectric spectrometer equipped with a 150 W xenon arc lamp combined with a potentiostat (Instytut Fotonowy) in a Teflon™ cuvette with a quartz window. All measurements were carried out in 0.1 M KNO_3_ using a three-electrode configuration with TiO_2_ NTs, a Pt wire and a saturated calomel electrode (SCE) serving as the working, counter, and reference electrodes, respectively. The photoanode was illuminated with monochromatic light in a range of 300–600 nm (increments of 10 nm) at potentials from −600 to 1000 mV (step size 100 mV) *vs.* SCE. Unless otherwise stated, the electrolyte was purged with Ar for 20 min before the measurements were taken. The photocurrent maps (photocurrent as a function of incident light wavelength and polarization potential) were obtained directly using PhotoelectricGUI software (Instytut Fotonowy). The recorded photocurrent values were then converted to incident photon to current efficiency (IPCE) in accordance with the following equation:2
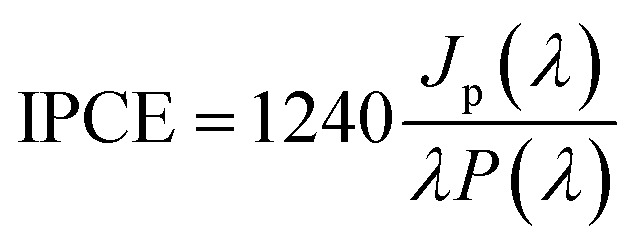
where *J*_p_(*λ*) is the photocurrent density recorded at a particular wavelength (*λ*) and *P*(*λ*) is the power density of the incident light at a particular wavelength (*λ*).

## 3. Results and discussion

**Fig. 2:**
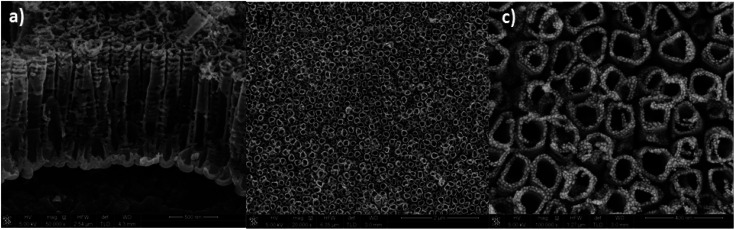
SEM images of a TiO_2_ nanotube layer formed at 25 V and annealed at 650 °C in air with a 0.01 mg cm^−2^ Au deposit: (a) cross-section, ((b) – low, (c) – high magnification) top-view.

Fig. 2 shows SEM images of TiO_2_ NTs formed at 25 V, heat-treated at 650 °C, and then loaded with evaporated Au (∼0.01 mg cm^−2^). The morphological observations revealed that the Au NPs are located on the tops and side walls of the nanotubes wich are ∼110 nm in diameter^27^ (Fig. 2a and b) and form rings around the tubes (Fig. 2c) that still reflect the original topography. As a result of gold evaporation at a high vacuum (10^−8^ mbar), the spherical plasmonic nanoparticles were distributed homogenously in the TiO_2_ nanotube layer.

**Fig. 3:**
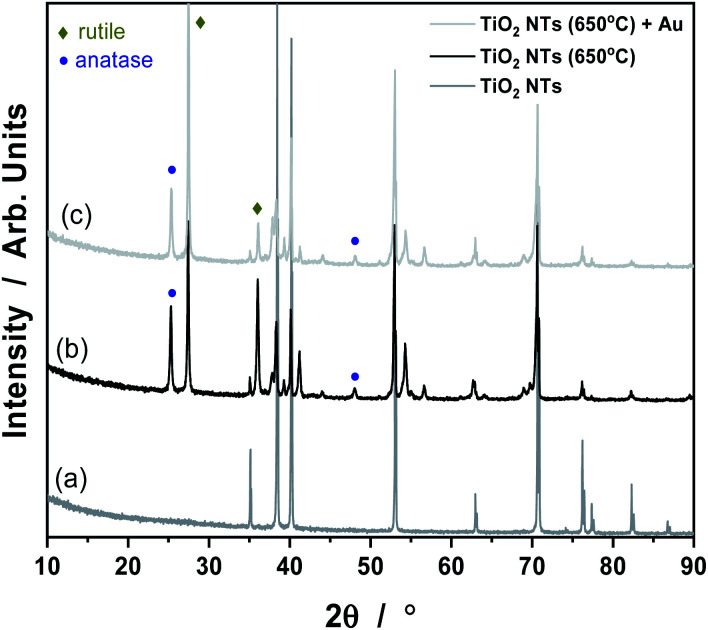
XRD patterns for TiO_2_ NTs in the as-received state (a), annealed at 650 °C (b) and heat treated + 0.01 mg cm^−2^ Au deposit (c).

It is known that anodization of TiO_2_ NTs leads to the formation of an amorphous phase of TiO_2_ material, which can be easily transformed into an anatase or rutile crystal structure under the influence of temperature.^30^ Our results from the XRD analysis all the samples tested are given in Fig. 3. The XRD pattern of TiO_2_ nanotubes in the as-received state sample did not reveal any TiO_2_ phases other than reflections from the Ti metal (a). Things were different for the samples annealed at 650 °C (b) and with Au deposit (0.01 mg cm^−2^) (c). In the (b) and (c) patterns, anatase and rutile signals are visible, which suggest the occurrence of a phase transition of anatase to rutile at this temperature.^27,31^ As shown in Fig. 3, there are two main characteristic peaks at 2*θ* = 25, 48° which indicates the anatase phase (ICSD: 154604) as well as peaks at 2*θ* = 27, 36° which corresponds to the rutile phase (ICSD: 39172). The XRD pattern of the Au/TiO_2_ NTs/Ti sample showed that, the strongest Au peak may have been masked by the Ti substrate peak at 2*θ* = 38 and 44°. However, the weaker peaks corresponding to metallic Au at 2*θ* values of about 64 and 77° are also not visible due to the presence of Ti oxides. Such results suggest that, this may be due to the low concentration of Au nanoparticles deposited on the TiO_2_ nanotubes, possibly below the detection limit of the XRD analysis.

**Fig. 4:**
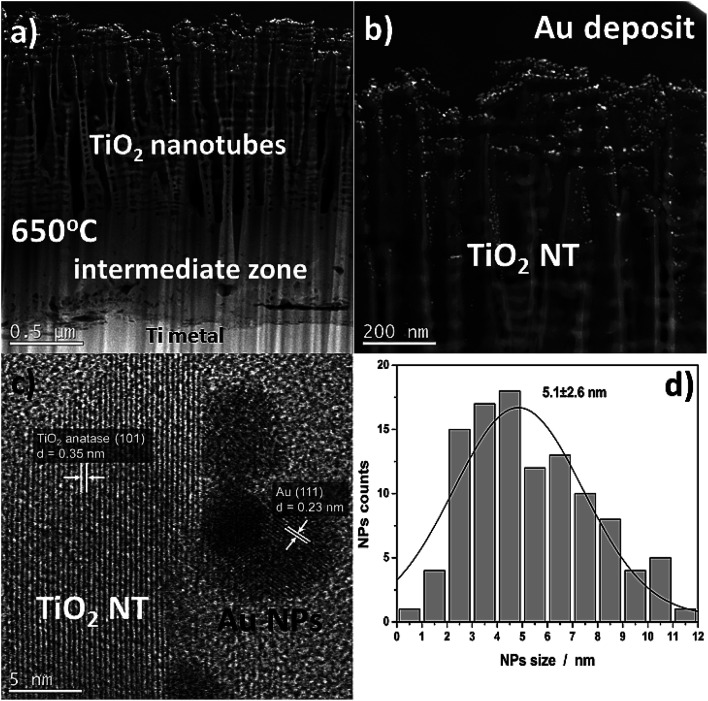
Cross-sectional view of annealed TiO_2_ NTs at 650 °C (25 V) with a 0.01 mg cm^−2^ Au deposit (a and b) HAADF-STEM images, (c) high-resolution STEM images showing a lattice spacing of TiO_2_ NTs and Au NPs, (d) histograms (nanoparticle counts *vs.* nanoparticle diameter).

More detailed information about the morphology and distribution of the deposited metal nanoparticles was provided by STEM investigations of the nanoporous cross section layer, Fig. 4. Three distinct domains can be distinguished within the cross-section (Fig. 4a): titanium oxide nanotubes (anatase + rutile), an interphase region (a compact TiO_2_ layer – mainly the rutile phase), and the titanium substrate.^26,28^ The formation of an intermediate zone was a result of a consolidation effect due to the nanotubes sintering with the metallic substrate during the heat treatment at 650 °C in air. The HR-STEM observations showed that after heat treatment at 650 °C, the TiO_2_ nanotubes have a partially anatase structure (Fig. 4c), which was confirmed in detail in our earlier work.^27^ However, it should be taken into account that the results presented in the HR-STEM images are only of a local nature, and the TiO_2_ nanotubular layer is a mixture of anatase and rutile phases. This is demonstrated by the XRD structural studies, see Fig. 3. The high-resolution investigations also showed that the Au NPs may occupy locations on the nanotube walls as deep as 600 nm from the top of the oxide layer (see Fig. 4b). Almost all of the nanoparticles were separated one from another. The HR-STEM images suggest that the interplanar distance between neighboring crystallographic planes is about 0.35 nm and 0.23 nm, and corresponds to the TiO_2_ anatase (101) and Au (111), respectively. A size distribution analysis of the Au NPs obtained from STEM investigations (Fig. 4d) indicates Gaussian-type behavior with maximum at 5.1 nm.

**Fig. 5:**
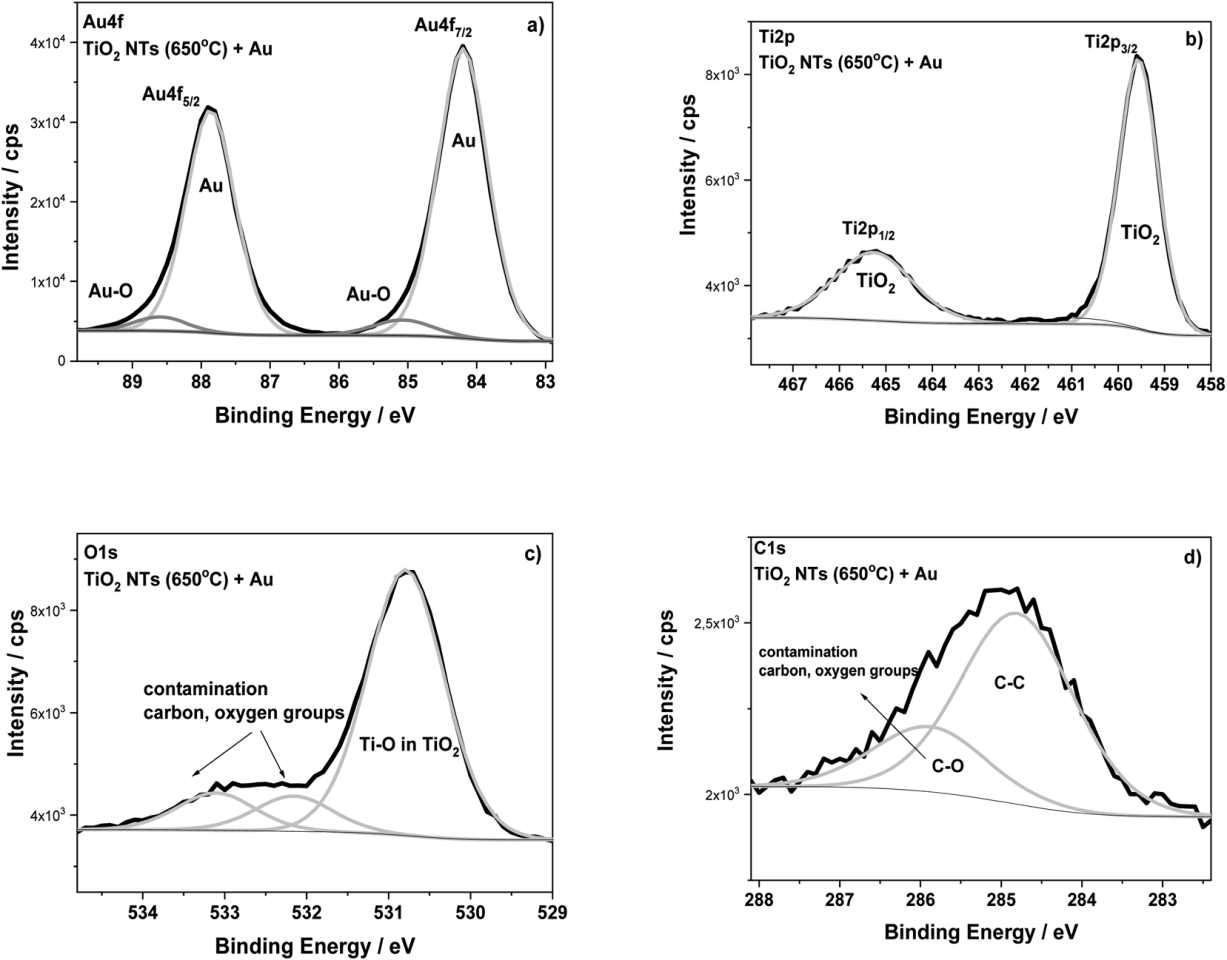
XPS high-resolution spectra for TiO_2_ NTs annealed at 650 °C in air with a 0.01 mg cm^−2^ Au deposit: (a) Au 4f, (b) Ti 2p, (c) O 1s and (d) C 1s regions.

The surface composition of the TiO_2_ NTs annealed at 650 °C in air (3 h) and after the deposition of 0.01 mg cm^−2^ Au were analyzed using XPS (Fig. 5). The HR XPS spectra for the Au, Ti and O elements indicate the presence of gold (Au 4f_7/2_ – 84.2 eV) on the top of nanotubes (Ti 2p_3/2_ – 459.6 eV; O 1s – 530.8 eV). The main signals of these spectra are characterized in Table 1. The HR spectra of C 1s and O 1s indicate the presence of carbon, and also oxygen functional groups, which correspond to typical surface contamination after exposure of the sample to air (see Fig. 3).^18,32^ The XPS data presented in Table 1 clearly show the BE of both Ti 2p and O 1s spectra recorded for TiO_2_ NTs sample to be shifted when Au was deposited. For the sample with the Au deposit, a positive shift in the Ti 2p_3/2_ (0.7–0.8 eV) and O 1s (0.6–0.7 eV) signals was registered in relation to both the as-received and annealed TiO_2_ NTs. This effect is probably associated with a strong metal–support interaction between the Au NPs and the TiO_2_ nanotubes.^6^ This supposition is confirmed by the BE position of the Au 4f_7/2_ core level spectrum (84.2 eV), which exhibits a positive shift in relation to the corresponding spectrum recorded for the pure Au foil (83.9 eV) (see Table 1). The observed shifts in Ti and Au could be caused by the formation of active places where negatively charged Au NPs are physically deposited on the TiO_2_ substrate without any chemical bonding between them.^17^ Such places could act as co-catalysts that facilitating electron–hole separation, where the activity strongly depends on particle size, geometry and the interspacing of the Au NPs.^1,24^ Moreover, it is known that the presence of the gold signal at the binding energy of around 85.0 eV may suggest the formation of Au–O (Au^1+^) bonds, which are important active places in catalytic processes, for example in CO conversion,^33^ see Fig. 5a. However, it should be noted here that the literature data usually show in this case a negative shift in the Au 4f spectra.^6,17,32^ This is connected with an electron transfer from oxygen vacancies in the TiO_2_ lattice to the Au. However, for our samples annealed at 650 °C, the formation of oxygen vacancies on the TiO_2_ NTs, should be considered together with the anatase–rutile phase transformation, because above 500 °C the rutile phase starts forming.^14^ Wen *et al.* observed a stronger binding of Au with the oxygen vacancies on the rutile surface as a result of an increase in the heat treatment temperature.^4^ Moreover, the literature studies have shown that the deposition of Au nanoparticles on a reduced rutile TiO_2_ (110) surface should result in a strong absorption of Au in the oxygen vacancies since the excess electron density in these sites can be donated to Au atoms.^32^ This causes the Au binding energy to shift towards lower values, as we observed during our research. Thus, the core level shifts in the XPS spectra are consistent with an electron transfer from titania to Au leading to electron-rich Au, as Goodman postulated for Au/TiO_2_ model catalysts^11,12^ but which are also dependent on the amount of low-coordinated surface metal atoms. This kind of SMSI effect, manifested by a positive shift of Au BE, is consistent with the present XPS results.

**Table 1:** XPS data evaluated from the deconvolution of the Ti 2p, O 1s and Au 4f spectra recorded at the surface layer of TiO_2_ NTs as prepared, annealed at 650 °C in air, and decorated with Au

Samples	Binding energy/eV			Au at%	Chemical state
	Ti 2p_3/2_	O 1s	Au 4f_7/2_		
TiO_2_ nanotubes as-received	**458.8**	**530.2**	—	—	**TiO**_**2**_
TiO_2_ nanotubes annealed at 650 °C	**458.9**	**530.1**	—	—	**TiO**_**2**_
TiO_2_ nanotubes annealed at 650 °C + 0.01 mg cm^−2^ Au	**459.6**	**530.8**	**84.2**	**31.2**	**TiO**_**2**_ **Au metal**
Reference data	**458.8**	—	—	—	**Ti in TiO**_**2**_ **(ref. 34)**
			**83.9**		**Au foil_ref. (this work)**
			**84.0**		**Au ref. 34**

**Fig. 6:**
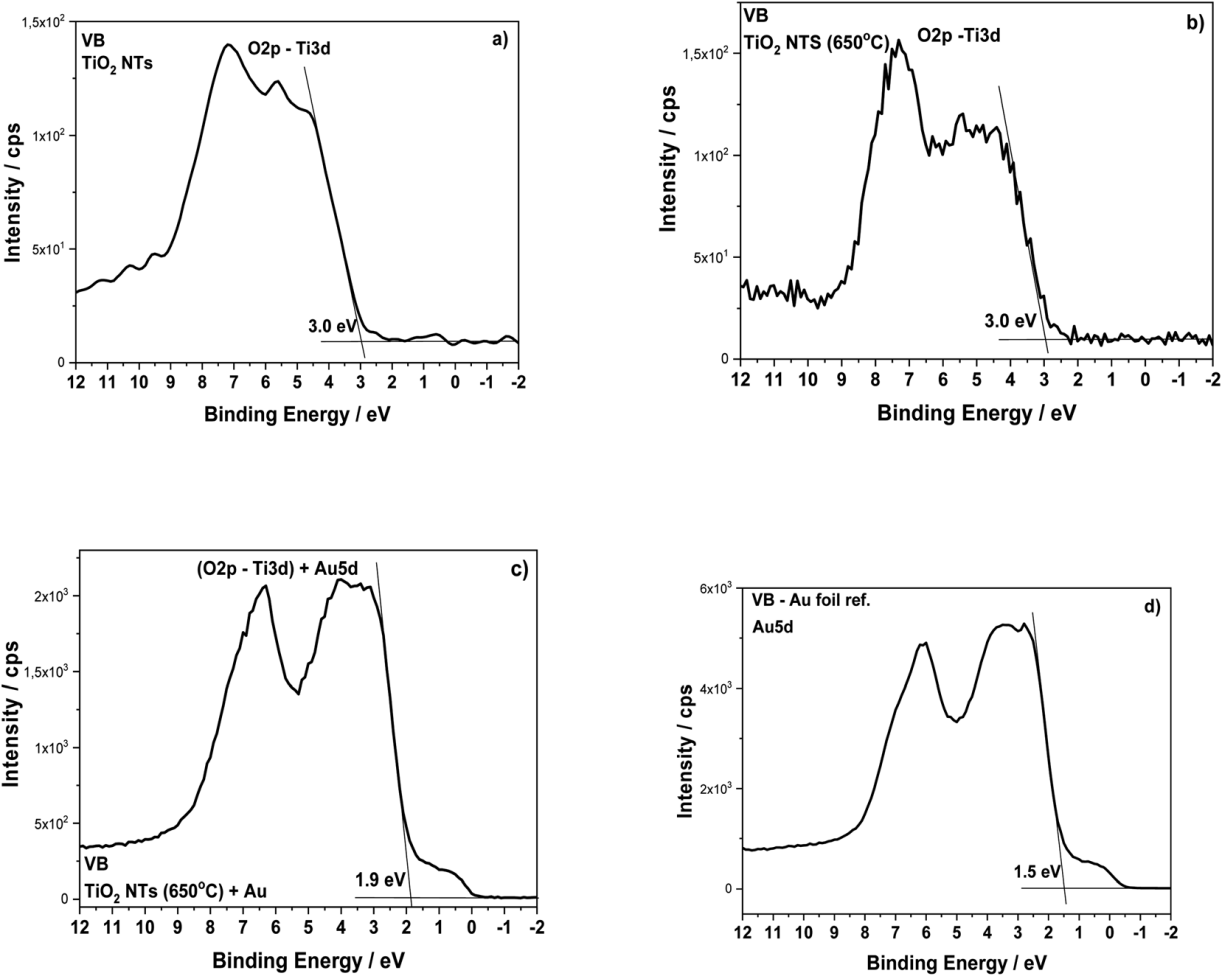
Valence band XPS spectra for the TiO_2_ NTs in as-received state (a), after heat treatment at 650 °C in air (b), after deposition of 0.01 mg cm^−2^ Au (c) and Au foil (reference spectrum) (d).

The structural transformation of the TiO_2_ NTs induced by the annealing procedure is well visible in the valence band (VB) XPS spectra (see Fig. 6). From the results presented in this work and our earlier works,^26–28^ we know that the structure of TiO_2_ NTs evolves from the amorphous phase in the as-prepared state to the anatase/rutile phase under annealing. The VB of the TiO_2_ NTs shows two peaks: a broad one centered at ∼5 eV and a narrow one at ∼7 eV, which correspond to the Ti 3d and O 2p orbitals, respectively. The VB maximum binding energies were determined by a linear extrapolation of the low binding energy valence band emission edge.^15,35^ Thus, the estimated band gap energy for both TiO_2_ NTs was ∼3.0 eV. The deposition of 0.01 mg cm^−2^ Au on the top and walls of the nanotubes after heat treatment clearly altered the shape of the VB XPS spectrum. The measured spectrum is similar to the pure gold valence band, and its character is mainly due to the presence of the Au 5d band. This is caused by a large number of Au NPs (31.2 atomic%, see Table 1) which decorate the tops of the nanotubes^32,36^ and their walls even to a depth of 600 nm (see, Fig. 2). Comparing the XPS-VB spectra for the Au–TiO_2_ NTs sample in relation to pure Au, some changes in the shape of the VB spectrum and a change in the determined value of the band gap energy can be seen. Thus, this observation could be further proof for the existence of an SMSI effect in the AuNPs/TiO_2_ NTs sample.

**Fig. 7:**
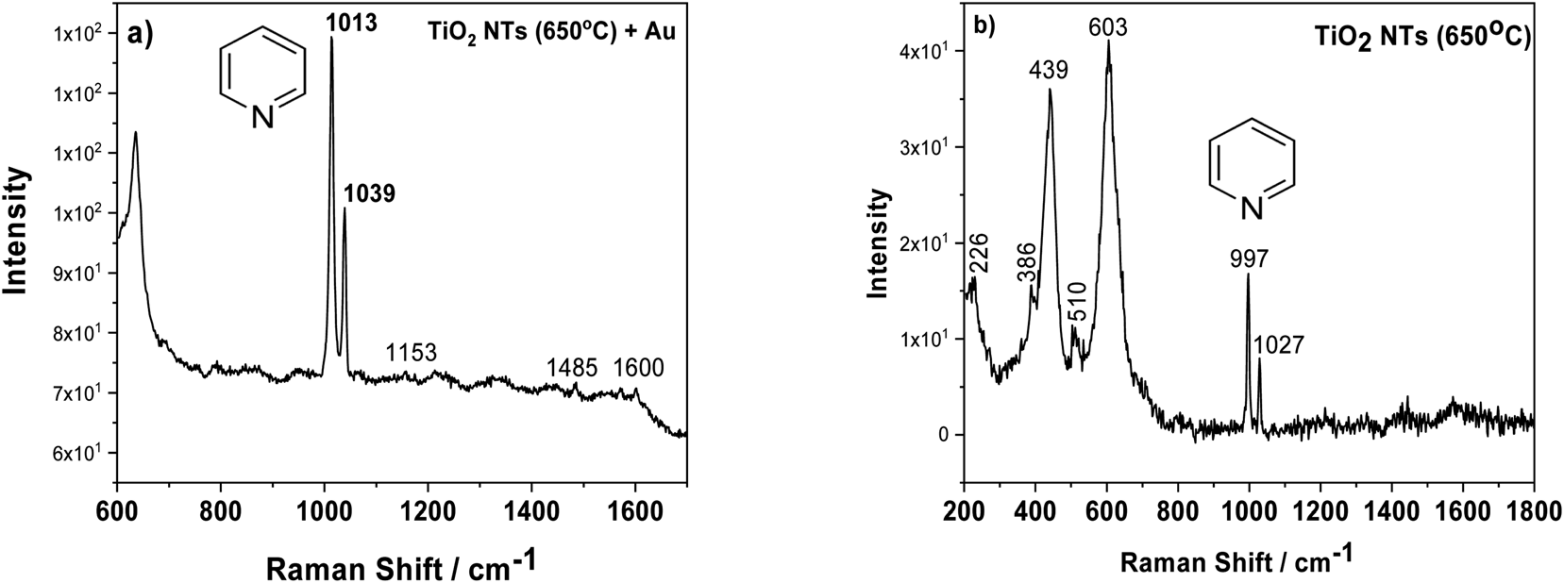
SERS spectra of adsorbed pyridine (5 × 10^−2^ M) on AuNPs/TiO_2_ NTS (a) and annealed tubes at 650 °C (b).

Another factor which may influence the photocatalytic properties of TiO_2_ nanotubes with an Au deposit is localized surface plasmon resonance (LSPR). To check this effect, we employed surface enhanced Raman spectroscopy (SERS), because densely-packed TiO_2_ NTs coated with Au NPs should act as nanoresonators. Fig. 7a shows the SERS spectrum of pyridine adsorbed on AuNPs/TiO_2_ NTs, where the characteristic bands at 1013 and 1039 cm^−1^ for this molecule are clearly visible. These bands originate from the pyridine symmetric ring deformation modes (*ν*_1_) and (*ν*_12_), respectively.^37,38^ These bands were very poorly visible for the SERS spectrum recorded on the annealed substrate at a temperature of 650 °C without gold nanoparticles, Fig. 7b. In this case, the peaks originating from the rutile structure at the 439 and 603 cm^−1^ (ref. 31) were the main bands. Moreover, it was also possible to observe weak bands from the anatase (226, 386, 510 cm^−1^).^31^ Comparing both SERS spectra, it can be seen that the intensity of the pyridine signal for the sample with Au is about 10 times higher than that of the pure substrate heated to a temperature of 650 °C. This means that the enhancement effect is related not only to the presence of the probe molecule but also to the gold nanoparticles. Moreover, the SERS spectrum measured using Au nanoparticles deposited on TiO_2_ NTs was characterized by other vibrational bands, in particular at ∼1600 cm^−1^ (*ν*_8a_) having A1 symmetry.^37,38^ Based on the literature, it can be assumed that pyridine in such a configuration interacts with the Au clusters^38^ due to the LSPR effect. The Raman signal enhancement is mainly caused by electromagnetic field enhancement (EM), where the “hot-spots” play a crucial role in chemical enhancement (CE), where the surface chemistry and electronic properties of the SERS platform are the most important. “Thus, the chemical enhancement is contributed from the chemisorption interaction, the photon-driven charge transfer (CT) between adsorbate and SERS substrate, and the coupling effect between the electron–hole pair and adsorbed molecule”.^38^ In our case, both mechanisms can have a significant role in amplifying the SERS signal, taking into account the specific morphology of the sample (the formation of the suitable gaps and cavities serving as surface plasmon resonators significantly increasing the intensity of the electromagnetic field) and a strong metal support interaction effect.

**Fig. 8:**
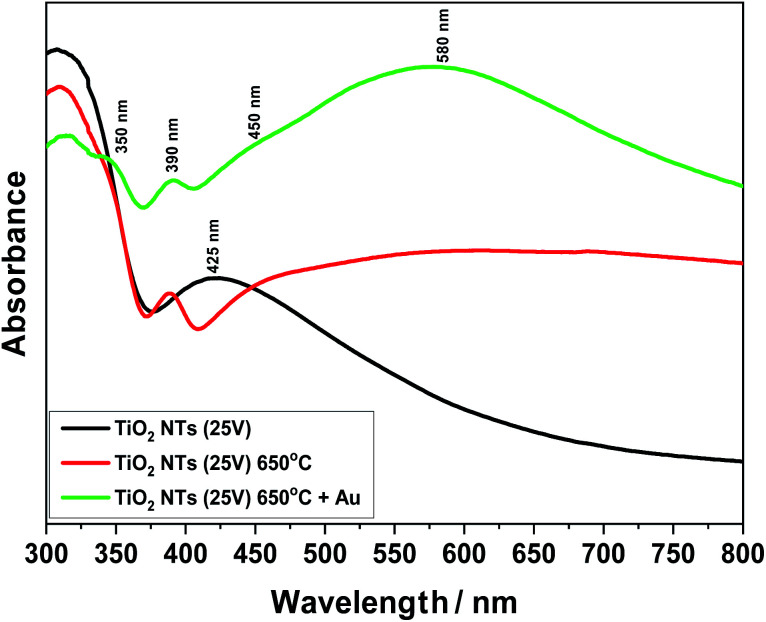
UV-Vis spectra for TiO_2_ NTs (25 V): in the as-received state (unheated); after heat treatment at 650 °C; after heat treatment and deposition of 0.01 mg cm^−2^ Au on the top of nanotubes.

Fig. 8 presents UV-Vis spectra for unheated (as-received state) and annealed samples at 650 °C. Furthermore, the effect of the 0.01 mg cm^−2^ Au deposit on light absorption from the infrared to visible light range is also shown. Comparing these spectra, a change in the structure of the titanium oxide (TiO_2_ NTs) from an amorphous to a crystalline phase under the influence of temperature can be observed. For the sample annealed at 650 °C, a clear sharp absorption peak can be seen at about 390 nm, which is characteristic for the rutile phase.^39,40^ Taking into account our previous structural studies (HR-STEM, XRD),^26–28^ we know that the rutile phase appears at the interface between the TiO_2_ nanotubes and the Ti metallic substrate – the transition zone visible in Fig. 4a. Moreover, the annealing process at 650 °C may lead to the appearance of local changes in the form of rutile phase precipitates in the anatase matrix. In the case of the unheated sample (as-received state), a relatively wide absorption peak in the range of 380 to 480 nm is well visible, which corresponds to the structure of pure TiO_2_.^17,19^ After the deposition of gold on the annealed samples in a range from 470 to 700 nm, an additional absorption peak associated with Au appears. The maximum of this broad peak is at 580 nm. The UV-Vis spectra of the sample with the Au deposit also revealed further weak maximums at around 350 and 450 nm. Probably, the peak at 350 nm can be attributed to absorption due to the interband transition in the Au NPs, as suggested by Noothongkaew,^17^ while the peak at 450 nm can be associated with defects in the titanium oxide crystal structure, as observed by Kato *et al.*^39^ and Khan *et al.*^41^ Moreover, a clear plasmon resonance shift for the sample with Au NPs (60–70 nm) is clearly visible (which corresponds to energy ∼0.2 eV) relative to the typical gold plasmonic peak position at 520 nm.^42,43^ This is related to two factors: the impact of nanoparticle size and shape^1,4,32,44^ and the strong interaction between the Au NPs and the TiO_2_ nanostructured substrate – the SMSI effect. Naldoni *et al.*^1^ postulated that this factor may affect the mechanism of photocatalytic reaction, where the PRET effect becomes the dominant mechanism in relation to the transfer of so-called hot electrons from the valence band (VB) to the conduction band (CB). Thus, the enhanced visible light absorption peak could be ascribed to an increased LSPR effect of the Au nanoparticles,^22^ which significantly increases the intensity of the UV-Vis spectra in a range of from 470 to 700 nm.

**Fig. 9:**
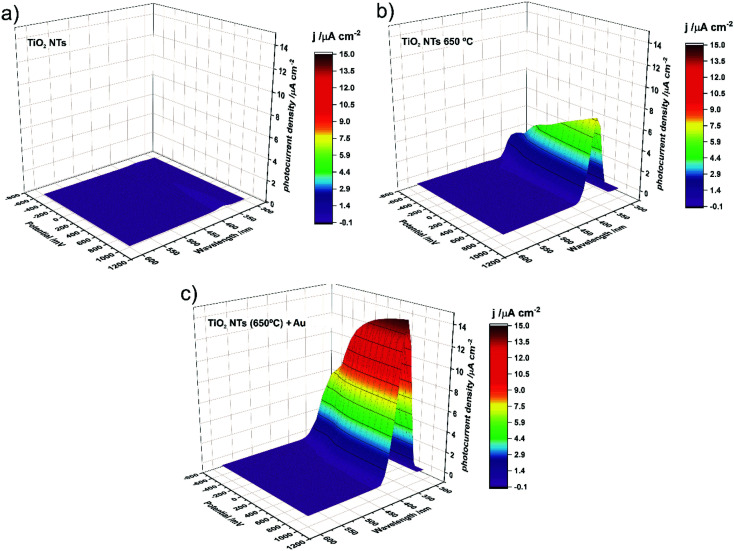
Photocurrent density as a function of incident light wavelength and applied potential recorded for as-anodized TiO_2_ NTs (a); TiO_2_ NTs annealed at 650 °C (b); and TiO_2_ NTs annealed at 650 °C decorated with Au NPs (c).

Photocurrent density maps recorded for the as-received and thermally-treated “bare” TiO_2_ NTs, as well as for the annealed TiO_2_ NTs covered with Au NPs, are shown in Fig. 9a–c, respectively. It is clear from Fig. 9a that the as-anodized TiO_2_ NTs exhibit an almost imperceptible photoresponse even in the UV region (a maximum photocurrent density of <0.2 μA cm^−2^ was observed for *λ* = 360 nm). This is because in amorphous nanotubes most of the photocurrent is generated in their bottom part and the contribution of the tube wall is negligible due to the high number of defects which are responsible for a high rate of carrier recombination.^45,46^ The forty-fold increase in the maximum photocurrent density (to *ca.* 7.7 μA cm^−2^ for *λ* = 370 nm) after annealing in air at 650 °C is a result of the conversion of the amorphous TiO_2_ to photoactive crystalline phases (anatase and a mixture of anatase and rutile, see Fig. 3 and 4) during the thermal treatment.^45–47^ As can be seen in Fig. 9c, the decoration of annealed TiO_2_ NTs with Au NP resulted in a further doubling of the maximum photocurrent density (to *ca.* 14.7 μA cm^−2^ for *λ* = 360 nm).

It should be noted at this point that the stability of the tested system practically does not change over time. After another test, after 23 days, it turned out that the photoresponse of the system was at the same level of about 15 μA cm^−2^. Moreover, additional SEM observations of the surface of the examined system after the photoelectrochemical tests revealed that the surface morphology did not change significantly. We still observe gold nanoparticles coating the TiO_2_ nanotubes with no change in size and distribution with respect to the surface shown in Fig. 2.

**Fig. 10:**
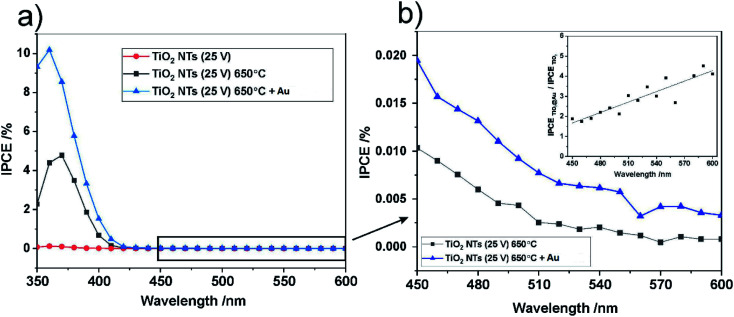
IPCE spectra at *U* = 1 V *vs.* SCE for all studied photo-anodes in the UV-Vis range (a) and IPCE spectra in the visible range for annealed TiO_2_ NTs, non-decorated and decorated with Au NPs (b). The inset in (b) shows the ratio of the IPCE of TiO_2_ NTs – Au NPs to the IPCE of the “bare” TiO_2_ NTs as a function of incident light wavelength.

For further insight into the observed effects, the IPCE (incident photon current efficiency) values at the potential of 1 V *vs.* SCE were calculated for all the photo-anodes studied; the results are shown in Fig. 10.

The spectra obtained confirm the most significant photoconversion in the UV range independently of the sample type, and it is clear that the highest IPCE values were achieved for the TiO_2_ NTs decorated with Au NPs. This enhancement of the photoactivity of TiO_2_ NTs in the UV range can be explained in terms of the plasmonic-induced resonant energy transfer (PRET) of the Au NPs, resulting in enhanced UV light absorption and charge carrier transport due to the strong electric field amplification near the TiO_2_/Au interface (LSPR and SMSI effect). This means that the presence of Au NPs promotes the absorption *via* the band gap transitions of TiO_2_.^48^ A detailed inspection of the IPCE spectra revealed that the Au NPs also enhanced the photoconversion of TiO_2_ NTs in the visible range (Fig. 10b). Although no well-defined peaks can be observed between 450 nm and 600 nm, it can be seen that the IPCE amplification level (defined as the ratio of the IPCE value observed for the photoanode modified with Au NPs to the value recorded for the “bare” TiO_2_ NTs) gradually increased along with the wavelength of the incident light (see inset in Fig. 10b). This is in agreement with the shape of the SPR peak in the absorption spectra. Therefore, it can be supposed that the SPR generation of hot electrons can also occur in the case of Au NPs-modified TiO_2_ NTs under visible light irradiation. Nevertheless, the photo-electrochemical measurements definitely confirmed that the PRET effect is predominant over the hot electron transfer in the materials studied.

**Fig. 11:**
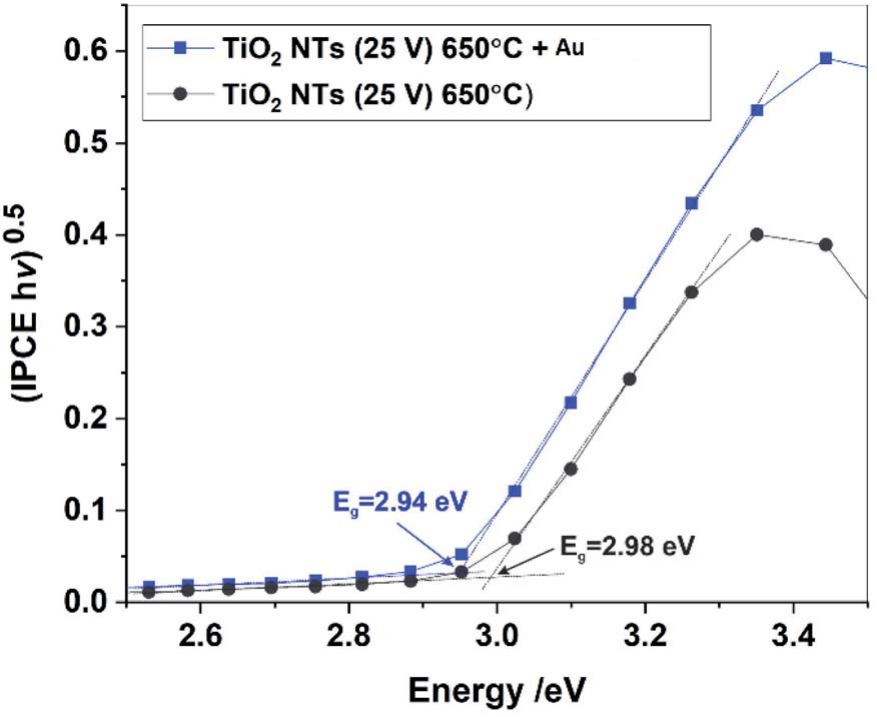
(IPCE *hν*)^0.5^*vs. hν* plots (Tauc plots) for annealed TiO_2_ NTs without and with Au NPs.

The band gaps of the annealed TiO_2_ NTs (both with and without Au NPs) were also estimated from (IPCE *hν*)^0.5^*vs. hν* plots by extrapolating the straight region to the baseline (see Fig. 11). It was confirmed that the decoration of TiO_2_ NTs with Au NPs results in a slight but noticeable decrease in the *E*_g_ of the photoanode. Unfortunately, for the unheated nanotubes it was not possible to determine the *E*_g_ value due to the poor photo response of the system (see Fig. 9).

Therefore, based on the diffuse reflectance spectroscopy (DRS) method and the Kubelka–Munk calculations,^40,49^ the band gaps were determined for all the received materials, as summarized in Table 2. Moreover, Table 2 compares the values of the band gap energy determined by other methods based on XPS-VB and photo-electrochemical measurements.

**Table 2:** Band gap energy calculated based on the Kubelka–Munk, XPS-VB and photo-electrochemical methods for functionalized TiO_2_ NTs

Samples	UV-Vis/eV	XPS-VB/eV	Photo-electrochemical measurements/eV
TiO_2_ NTs (25 V)	3.3	3.0	Weak response
TiO_2_ NTs (25 V) 650 °C	3.3	3.0	∼3.0
TiO_2_ NTs (25 V) 650 °C + 0.01 mg cm^−2^ Au	3.2	1.9^a^	∼2.9

a XPS-VB, this value of band gap energy is related to Au; a similar value for Au was also estimated based on the Kubelka–Munk calculation (∼2.1 eV).

The small differences in band gap energy are associated with physical principles and the depth resolution characteristic for all the methods used. Nevertheless, the results are consistent. The observed differences in band gap energy are due to the fact that the subject of the our studies is substrates with a large surface development, which affects the dispersion of incident light. Therefore, the determined energies of the forbidden gaps are characteristic for TiO_2_ structures.^49^ This indicates that physically adsorbed Au NPs do not significantly modify the gaps in the band structure of TiO_2_. This is another factor that indicates that the PRET mechanism is dominant in the transfer of hot electrons supported by a plasmon-induced charge separation effect, which allows direct conversion of LSPR to electron flows and photo-electrochemical reactions.^50^

**Fig. 12:**
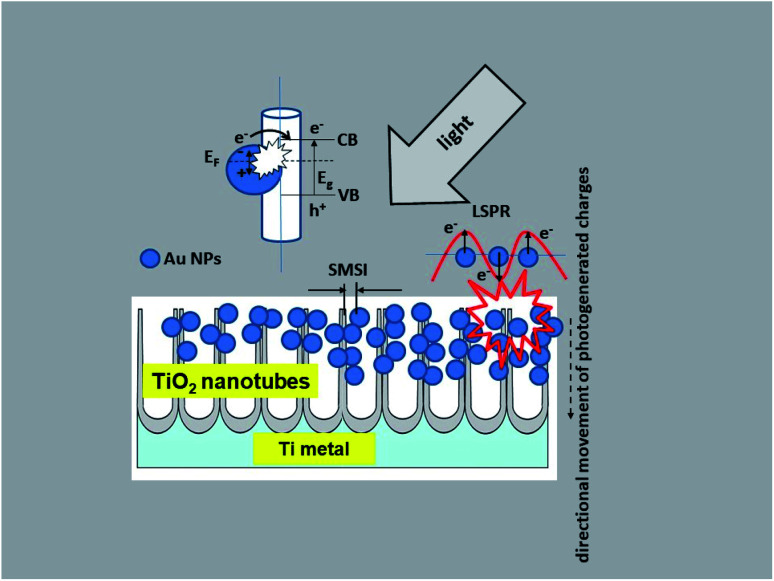
Schematic diagram of the mechanism of charge transfer (PRET and LSPR) in the Au NPs/TiO_2_ NTs system.

Taking into account the results of investigations presented in this paper the following charge transfer mechanism can be proposed for our samples, as shown in Fig. 12. It is generally known that the Fermi level of a noble metal is lower than that of TiO_2_. Therefore, electrons can be easily transferred from the oxide to metal nanoparticles deposited on its surface, which significantly reduces the possibility of electron–hole recombination. This usually leads to efficient charge separation and an increase in the photocatalytic reaction.^22^ This is related to the LSPR effect, where high-energy electrons (hot electrons e^−^) are generated, and to the possibility of concentrating the electromagnetic field in nanoscale volumes. The resulting heterojunction between the TiO_2_ oxide (nanotubes) and the gold nanoparticles leads to a rapid interfacial photo-generated electron transfer from TiO_2_ NTs to Au NPs after crossing the Schottky barrier, increasing the separation of e^−^/h^+^ photo-generated pairs.^1,20,22^ The efficiency of this process is strongly related to the size and distribution of the nanoparticles. Mainly small Au nanoparticles (below 10 nm) result in higher efficiency.^20^ This mechanism has been described in detail in the work of Naldoni,^1^ which demonstrated the influence of nanoparticle size on the SMSI effect, which increases the probability of generating charge carriers at the Au/TiO_2_ interface.

## 4. Conclusions

The use of Au NPs as a source of the strong absorption of visible light due to the LSPR and SMSI effects proved to be an effective way to improve the photocatalytic activity of AuNPs/TiO_2_ NTs samples. We can distinguish two categories of effects which overlap and lead to the formation of mixed sites at the metal/oxide interface as a result of electronic effects originating from the support^51^ and nature of the physically adsorbed metallic nanoparticles (structure, morphology, amount of low-coordinated surface metal atoms, size and shape of Au NPs).^51,52^ Such changes were clearly visible in the high-resolution (HR) and valence band (VB) XPS spectra, where positive shifts for the Ti 2p, O 1s, Au 4f and VB binding energies were found. A similar effect was also observed in the UV-Vis spectra and the photo-electrochemical measurments, where in addition to the characteristic positions for TiO_2_ and Au other absorption peaks associated with the structure of the nanotubes and the interaction of Au–TiO_2_ were observed. These changes generated a final effect in the form of an Au LSPR band shift towards the visible light region in the UV-Vis spectrum and an increase in the photocurrent density as a function of incident light wavelength. Thanks to these phenomena, we observed a huge increase in the photocurrent density value, from ∼0.2 μA cm^−2^ for the nanotubes in the as-received state to 14.7 μA cm^−2^ for the nanotubes with gold NPs. The IPCE (incident photon current efficiency) spectra also revealed that the Au NPs enhanced the photoconversion of TiO_2_ NTs in the visible range, without significantly changing the band gap energy of the titanium dioxide, which was about 3.0 eV. The absence of a clear change in the energy band gap suggests that the dominant effect responsible for the photoactivity of the obtained materials was PRET. Moreover, the results of our studied show that both nano size AuNPs and mixed anatase/rutile structure of AuNPs/TiO_2_ NTs samples are responsible for their photocatalytic activity.

## Author contributions

Marcin Pisarek: conceptualization – ideas; formulation or evolution of overarching research goals and aims. Project administration – management and coordination responsibility for the research activity planning and execution. writing – original draft – preparation, creation and/or presentation of the published work, specifically writing the initial draft (including substantive translation). Investigation – conducting a research and investigation process, specifically performing the experiments, or data/evidence collection (formation of TiO_2_ nanostructures). Mirosław Krawczyk: investigation – conducting a research and investigation process, specifically performing the experiments, or data/evidence collection (deposition process of Au NPs in UHV conditions). Andrzej Kosiński: resources – provision of study materials, reagents, materials, patients, laboratory samples, animals, instrumentation, computing resources, or other analysis tools. Visualization – preparation, creation and/or presentation of the published work, specifically visualization/data presentation. Marcin Hołdyński: investigation – conducting a research and investigation process, specifically performing the experiments, or data/evidence collection. Formal analysis – application of statistical, mathematical, computational, or other formal techniques to analyze or synthesize study data (SEM/STEM observations, XRD investigations). Mariusz Andrzejczuk: investigation – conducting a research and investigation process, specifically performing the experiments, or data/evidence collection (STEM observations). Jan Krajczewski: investigation – conducting a research and investigation process, specifically performing the experiments, or data/evidence collection (SERS measurements). Krzysztof Bieńkowski: investigation – conducting a research and investigation process, specifically performing the experiments, or data/evidence collection (UV-Vis measurements). Renata Solarska: writing – review & editing – preparation, creation and/or presentation of the published work by those from the original research group, specifically critical review, commentary or revision – including pre- or post-publication stages (UV-Vis measurements). Magdalena Gurgul: investigation – conducting a research and investigation process, specifically performing the experiments, or data/evidence collection (photoelectrochemical measurements). Leszek Zaraska: investigation – conducting a research and investigation process, specifically performing the experiments, or data/evidence collection. Writing – review & editing – preparation, creation and/or presentation of the published work by those from the original research group, specifically critical review, commentary or revision – including pre- or post-publication stages (photoelectrochemical measurements). Wojciech Lisowski: investigation – conducting a research and investigation process, specifically performing the experiments, or data/evidence collection. Writing – review & editing – preparation, creation and/or presentation of the published work by those from the original research group, specifically critical review, commentary or revision – including pre- or post-publication stages (XPS measurements).

## Conflicts of interest

There are no conflicts to declare.

## Supplementary Material
